# Conserved temperature requirements but contrasting responses to humidity across oviposition preferences in temperate grasshoppers

**DOI:** 10.1038/s41598-023-47789-z

**Published:** 2023-11-30

**Authors:** Tomáš Dvořák, Michal Knapp

**Affiliations:** 1 Department of Zoology, Faculty of Science, Charles University, Viničná 7, Prague 2, 128 00 Czech Republic; 2 Department of Ecology, Faculty of Environmental Sciences, Czech University of Life Sciences Prague, Kamýcká 129, Prague-Suchdol, 165 00 Czech Republic

**Keywords:** Ecology, Evolution, Physiology, Zoology

## Abstract

The right choice of an oviposition site is a crucial task for oviparous species without maternal care. In contrast to well investigated biotic factors, e.g., larval food preferences, parasitism, predation, and competition avoiding, abiotic factors affecting oviposition preferences in insects have been rarely investigated in comparative studies. To improve our current understanding of oviposition site selection in Orthoptera, we investigated the influence of substrate temperature and moisture on the oviposition behaviour of 14 temperate grasshopper species. Conspecific groups of adults were kept in arenas with simultaneous temperature and moisture gradients. For each ootheca produced during the experiment (n = 1192) we recorded its depth and local microclimatic conditions. Our results indicate that microclimatic oviposition preferences significantly differ among species, however, correlations between adult habitat preferences and microclimatic oviposition preferences were surprisingly weak. Even oligothermic species preferred substrate temperatures around 30 °C and some xerothermic species preferred higher humidity. The hypothesized tendency to place oothecae closer to the ground within grass tussocks under hot and dry conditions was confirmed. It is possible that species evaluate microclimatic conditions for oviposition in the context of occupied habitat, i.e., in a relative rather than absolute manner.

## Introduction

Searching for the optimal environment that allows for the successful development of eggs and subsequent offspring survival is one of the crucial issues faced by every oviparous ectotherm species without direct maternal care^1^. Most studies have focused on biotic factors associated with the optimal food availability for hatchlings or parasitism, predation, and competition avoiding^2–6^. Abiotic factors, such as moisture and temperature, have received significantly less attention^7–9^ and comparative studies among species are largely missing. In addition, many studies are focused on reptile oviposition site selection^10–12^, and there is only limited information on the most diverse and numerous group of ectotherms, insects^13,14^. With ongoing climate change and anthropogenic habitat degradation frequently affecting local moisture and temperature, understanding the role of microclimate on oviposition behaviour and preferences in insects has become an especially important research topic for ecologists and entomologists^15–17^.

Current theory on oviposition site selection in insects can be classified into two main hypotheses: (1) the environmental matching hypothesis postulates that oviposition preferences are associated with the overall xerothermophility (an affinity for dry and warm sites) of a species^7,13,18^, i.e., species inhabiting dry and warm habitats prefer to lay eggs in dry and warm microhabitats; (2) the insurance hypothesis postulates that all species prefer relatively humid substrate, independently of their habitat preferences, to avoid egg desiccation^9,19,20^. To test these hypotheses, multispecies comparative studies using standardized experimental designs are needed^13,21^. Unfortunately, the existing studies have mostly investigated a single species^9,18,20,22^ or compared two species with contrasting habitat requirements^7,19,23^. For a few species, intraspecific differences between various populations have also been investigated^24,25^. Despite a potentially strong synergic effect, moisture and temperature have been rarely investigated in parallel^26,27^. Experimental designs that combine independent gradients of both variables are needed to distinguish between independent and interactive effects of local temperature and substrate humidity.

Temperate grasshoppers from the subfamily Gomphocerinae represent an optimal model group for multispecies comparative studies. This subfamily contains a high number of morphologically uniform species with similar life histories^21^ and, at the same time, particular species strongly differ in their habitat preferences^28–30^. The limited existing knowledge indicates that grasshoppers follow the environmental matching hypothesis rather than the insurance hypothesis, i.e., their microclimatic oviposition preferences are correlated with species habitat preferences^7,21,31,32^. However, systematic investigations are needed to provide a more nuanced and unambiguous conclusion.

Within a suitable microhabitat, ovipositing females can further adjust their laying behaviour to meet optimal conditions for egg development^8,33^. For example, species using soil and grass tussocks for egg laying can manipulate oviposition depth (according to the vertical distribution plasticity hypothesis), as moisture commonly increases and temperature decreases with increasing depth^21,24,34^. In particular, it has been hypothesized that xerothermophilic grasshopper species lay eggs deeper into the substrate to avoid exposure to extreme temperatures and drought, while species inhabiting cold and moist habitats prefer shallow oviposition or grass tussocks to increase developmental temperature or to escape egg flooding^7,21^. However, for orthopterans, there is still only very limited evidence on inter- and intra-specific variation in this trait, as well as the overall variability in condition-dependent oviposition depth^21,24^.

This study investigated the oviposition preferences of 14 Central European Gomphocerinae grasshopper species using laboratory experiments that combined independent moisture and temperature gradients. The specific goals were to: (1) identify moisture, temperature, and depth preferences for egg-laying of all investigated species; (2) evaluate the oviposition preference hypotheses by comparing data from this study with adult habitat preferences investigated in our previous study (xerothermophility indices^30^); (3) assess whether grasshoppers modify their oviposition behaviour (laying depth) according to local microclimatic conditions.

## Results

### Oviposition preferences

**Figure 1:**
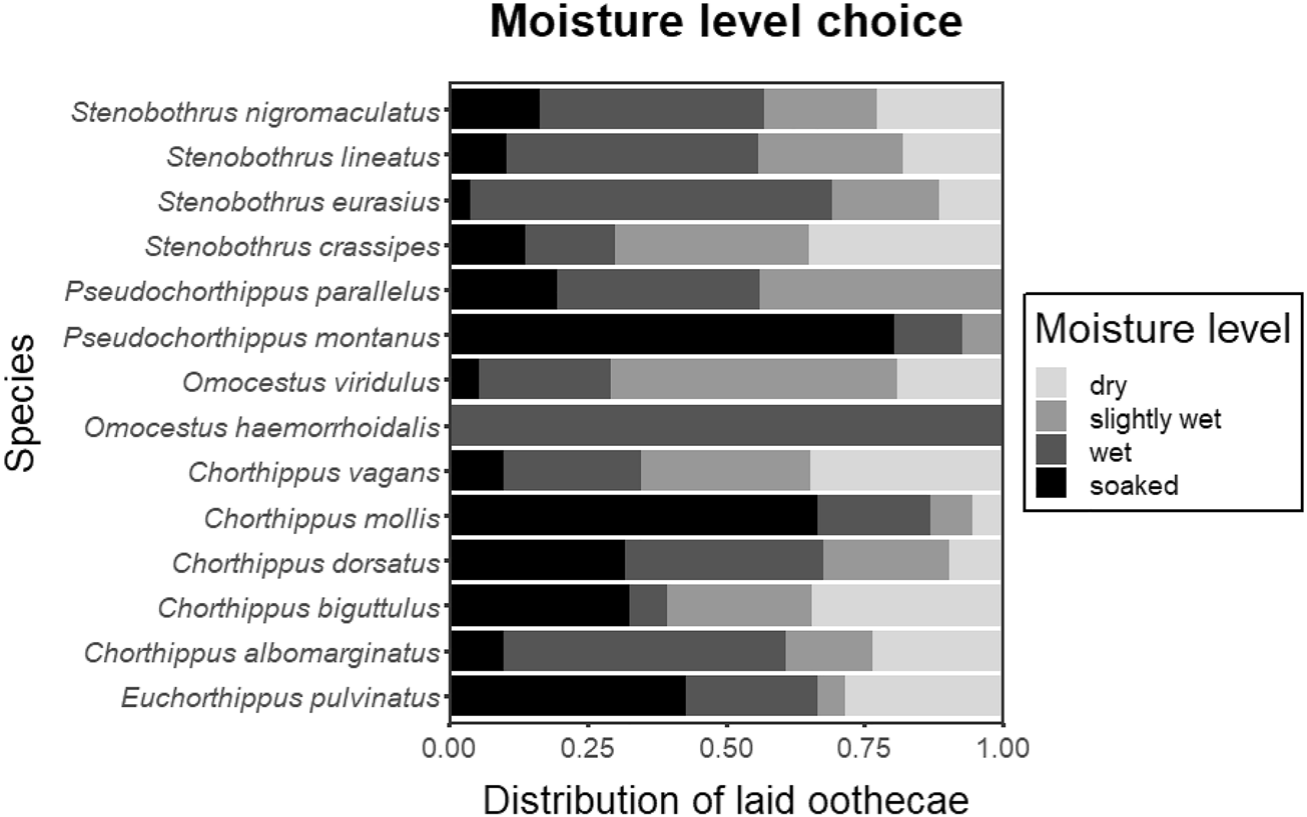
Species-specific distribution of oothecae in relation to humidity. Data for all species significantly differed from a random distribution (0.25:0.25:0.25:0.25).

A total of 1192 oothecae were produced by 280 females from the 14 investigated grasshopper species (Table 1). All species showed a non-random oviposition microhabitat selection with respect to moisture (Fig. 1), with *Stenobothrus nigromaculatus* being the closest to a random distribution of oothecae (χ^2^ = 10.7, df = 3, p = 0.013). The other extreme was represented by *Omocestus haemorrhoidalis*, which laid only 10 oothecae exclusively in the wet compartment. Three species preferred soaked substrate, six species preferred wet substrate and five species preferred slightly wet or dry substrate (Fig. 1; Table S1). Overall, across all species, wet substrate was the most preferred (contained 36% of all oothecae), followed by soaked (25%), slightly wet (22%) and dry substrate (17%). The strongest preference for moist substrate was recorded for *Pseudochorthippus montanus*, and the driest substrate was selected by *Chorthippus vagans* and *Stenobothrus crassipes*.

**Table 1:** List of investigated species and summary of their oviposition performance.

Species	Number of oothecae	Proportion of tussock-laid oothecae (%)	Index of xerothermophility
*Euchorthippus pulvinatus*	42	0	0.896
*Chorthippus albomarginatus*	51	2	0.336
*Chorthippus biguttulus*	160	0	0.518
*Chorthippus dorsatus*	145	100	0.375
*Chorthippus mollis*	54	4	0.900
*Chorthippus vagans*	92	12	0.960
*Omocestus haemorrhoidalis*	10	40	0.799
*Omocestus viridulus*	189	100	0.109
*Pseudochorthipus montanus*	41	0	0.021
*Pseudochorthipus parallelus*	41	32	0.291
*Stenobothrus crassipes*	80	51	0.780
*Stenobothrus eurasius*	26	88	1.000
*Stenobothrus lineatus*	183	100	0.777
*Stenobothrus nigromaculatus*	79	100	0.952

Number of laid oothecae per species and proportion of tussock-laid oothecae are shown. In addition, the xerothermophility index value obtained from our previous study^30^ is included for each species.

**Figure 2:**
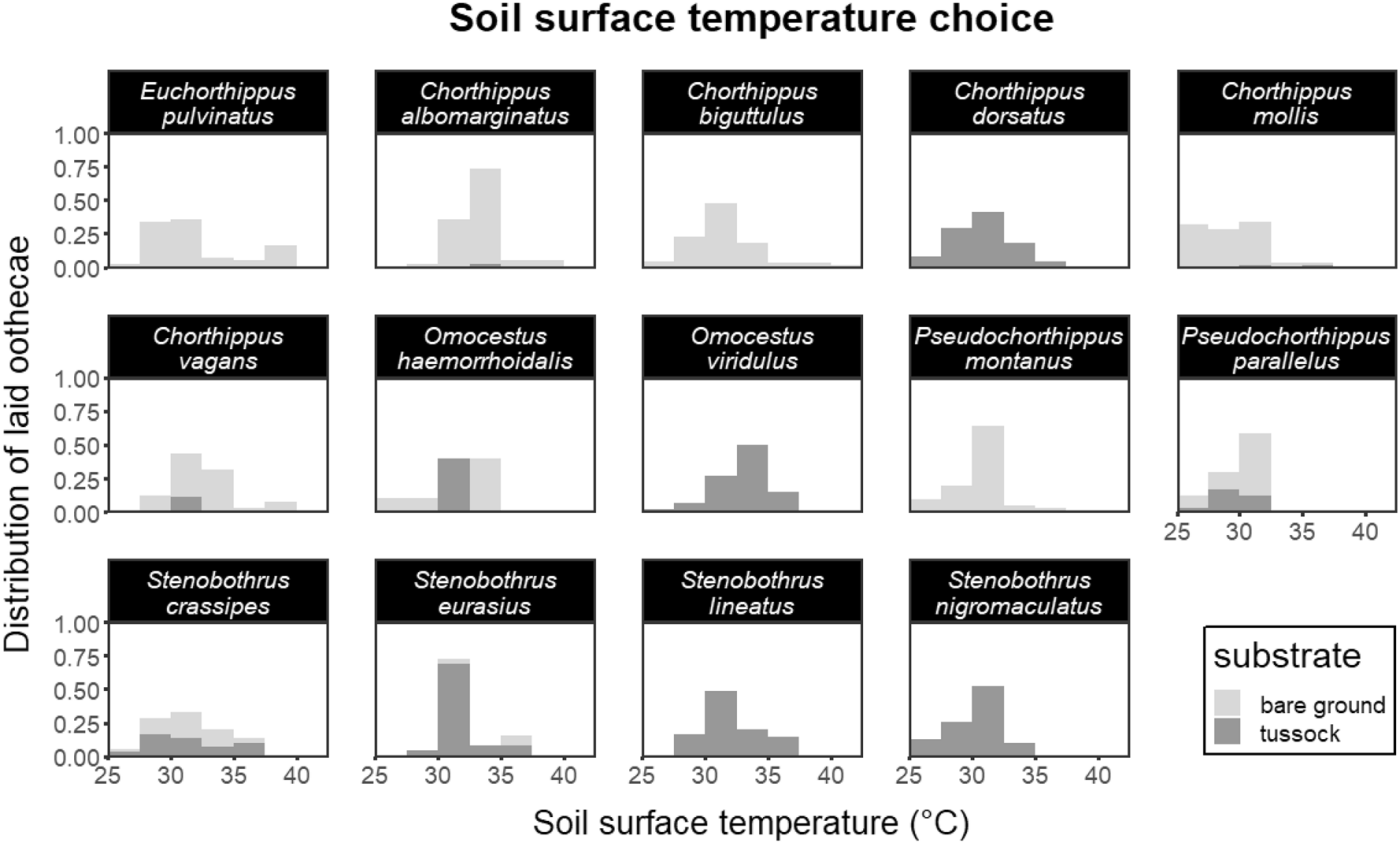
Species-specific distribution of oothecae in relation to temperature. Bare ground-laid and tussocks-laid oothecae are distinguished by colours (see the legend).

The highest average temperature for bare ground-laid oothecae was recorded for *Chorthippus albomarginatus* (33.2 °C), and the lowest for *Chorthippus mollis* (29.2 °C). Among tussocks-laid oothecae, the highest average temperature was observed for *Omocestus viridulus* (33.3 °C), and the lowest for *Pseudochorthippus parallelus* (29.6 °C). The preferred soil temperatures for oviposition are summarised in Table S1 and visualised in Fig. 2. Thermal preferences significantly differed between some species (bare ground-laid oothecae: F = 11.43, df = 9, p < 0.001; tussocks-laid oothecae: F = 14.47, df = 10, p < 0.001), however, there was also a large group of species with very similar thermal preferences (Table S1).

Vertical positions of oothecae ranged from 4 cm below ground to 5 cm above the soil surface (Table S1; Fig. S1). The deepest ootheca was buried by *Chorthippus vagans*, but the lowest mean depth was observed for *Euchorthippus pulvinatus* (2.37 ± 0.71 cm below ground). The species with the highest mean laying location was *Omocestus viridulus* with a mean of 1.34 ± 1.05 cm above the soil surface*.* There were significant differences in the vertical position of oothecae between the great majority of investigated species (bare ground-laid oothecae: F = 20.3, df = 9, p < 0.001; tussocks-laid oothecae: F = 36.58, df = 10, p < 0.001; Table S1).

### Oviposition site selection hypotheses

**Figure 3:**
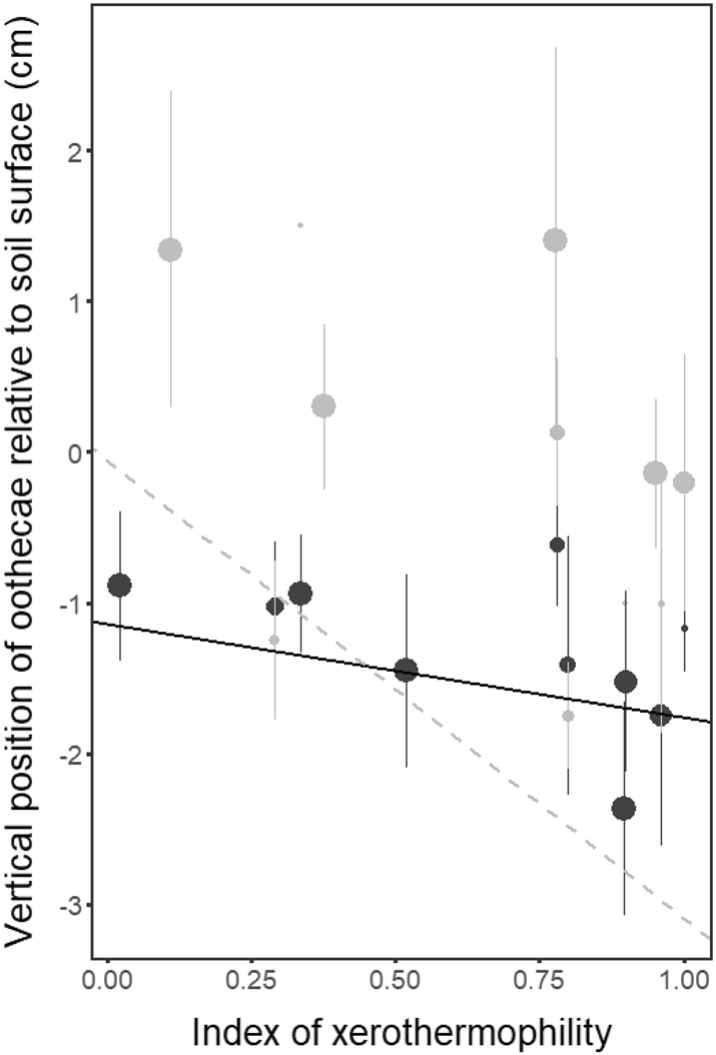
Relationship between vertical position of oothecae and species index of xerothermophility. Each dot represents mean vertical position of oothecae laid by one species (error bars represent ± SD). Bare ground-laid (dark grey) and tussocks-laid (light grey) oothecae are shown separately. Dot size represents species’ affinity to the given substrate type. The full line represents the phylogenetically corrected significant relationship between the vertical position of oothecae and the index of xerothermophility for tussocks-laid oothecae (p = 0.019). The dashed line represents the same but only marginally significant relationship for bare ground-laid oothecae (p = 0.079). The zero at the y-axis refers to the soil surface. Thus, positive values are above the ground, negative bellow the ground.

Results showed that there was no significant relationship between species-specific oviposition preferences (preferred moisture level, substrate temperature, and partially the vertical position of oothecae) and the index of xerothermophility for both bare ground-laid and tussock-laid oothecae, with the exception of the vertical position of tussocks-laid oothecae (Table 2a). Highly xerothermophilic species placed their oothecae significantly closer to the ground within grass tussock than less xerothermophilic species. Interestingly, the significance of this relationship was not confirmed for bare ground laying species despite the noticeable regression slope (Fig. 3). Phylogenetically-uncorrected models showed very similar results in the case of moisture and temperature. The only exception was the vertical position of oothecae, where the direction of the relationship was the same but the significance was switched between the bare ground and tussocks laying species (Table 2b). Our results for moisture strongly support the insurance hypothesis, as wet substrate was, in general, the most preferred substrate and the fewest number of oothecae were placed in the dry compartment across all the investigated species (Fig. 1).

**Table 2:** Effects of species xerothermophility on oviposition preferences.

(a) Substrate type	Dependent variable	Chi-sq	d.f.	p-value
Bare ground	Moisture	0.007	1	0.934
	Temperature	0.658	1	0.417
	Vertical position	3.081	1	0.079
Tussocks	Moisture	1.883	1	0.170
	Temperature	0.773	1	0.395
	Vertical position	5.502	1	**0.019**

(b) Substrate type	Dependent variable	Chi-sq	d.f.	p-value
Bare ground	Moisture	1.063	1	0.303
	Temperature	0.455	1	0.500
	Vertical position	4.180	1	**0.041**
Tussocks	Moisture	0.379	1	0.538
	Temperature	0.476	1	0.491
	Vertical position	0.884	1	0.347

Results of phylogenetic mixed-effects (a) and phylogenetically-uncoreccted (b) models investigating the relationship between the index of xerothermophility (independent variable) and species oviposition preferences for moisture, temperature, and ootheca vertical position (separate model was fitted for each response variable and substrate type). Bolded values indicate significant relationships.

Significant values are in bold.

**Figure 4:**
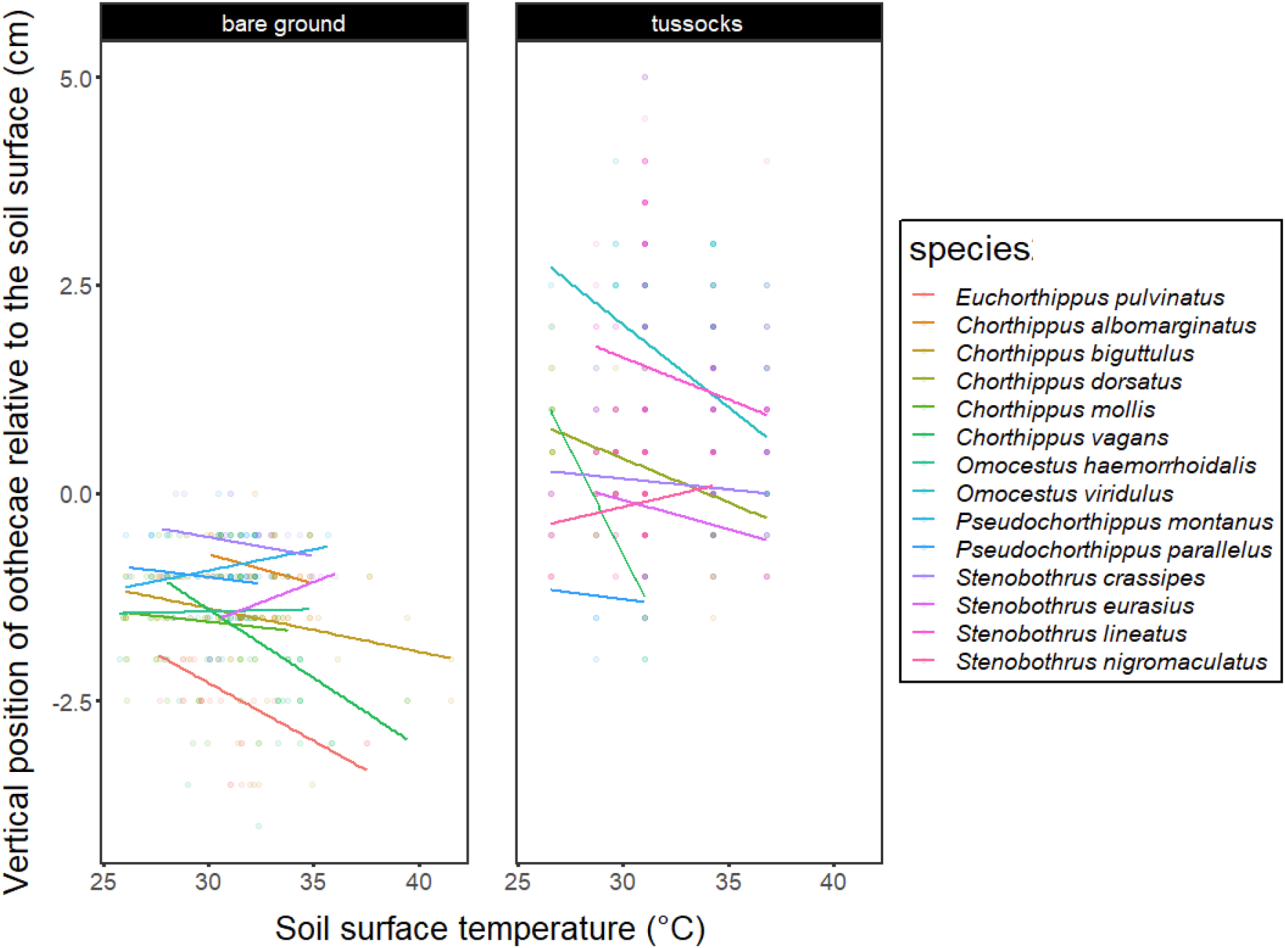
Effects of temperature on vertical position of oothecae. Separate regression lines are shown for each species. Each dot represents an individual ootheca (note that oothecae from different species placed to the same combination of conditions can overlap). The left graph shows bare ground-laid oothecae and the right shows tussocks-laid oothecae. The overall relationship (across species) between temperature and vertical position of oothecae was significant for tussock-laid oothecae (p = 0.014) and marginally significant for bare ground-laid oothecae (p = 0.071).

The vertical position of oothecae at the intraspecific level was significantly affected by moisture level and partially by temperature (Table 3a). Oothecae were placed shallower in bare ground or higher on tussocks with increasing local moisture (Figs. S2 and S3). Oothecae were placed significantly lower on tussocks and tend to be placed deeper in bare ground with increasing local temperature (Table 3a, Figs. 4 and S3). There was no significant interactive effects of local moisture and temperature on the vertical position of oothecae. Phylogenetically-uncorrected models performed practically identical compared to the corrected ones (Table 3b). Note that the above-described results represent overall patterns (across species) and patterns for particular species can be slightly different (for details see Figs. 4, S2 and S3).

**Table 3:** Effects of substrate moisture and surface temperature on vertical position of oothecae.

(a) Substrate type	Independent variable	Chi-sq	d.f.	p
Bare ground	Moisture	18.02	3	** < 0.001**
	Temperature	3.26	1	0.071
	Moisture: temperature	2.6	3	0.459
Tussock	Moisture	27.61	3	** < 0.001**
	Temperature	6.09	1	**0.014**
	Moisture: temperature	6.25	3	0.100

(b) Substrate type	Independent variable	Chi-sq	d.f.	p
Bare ground	Moisture	18.48	3	** < 0.001**
	Temperature	3.33	1	0.068
	Moisture: temperature	2.74	3	0.434
Tussock	Moisture	27.50	3	** < 0.001**
	Temperature	6.32	1	**0.012**
	Moisture: temperature	6.24	3	0.101

Results are reported separately for the phylogenetic mixed-effect (a) and phylogenetically-uncorrected (b) models.

Significant values are in bold.

## Discussion

All the investigated orthopteran species showed a non-random choice of moisture level, surface temperature, and vertical position when ovipositing. Interestingly, there was no relationship between preferred moisture or temperature during oviposition and species xerothermophility based on adult habitat preferences^30^. Instead, most species preferred higher levels of moisture and a relatively narrow range of temperatures. Nevertheless, there was a significant relationship between species xerothermophility and vertical position of oothecae laid in tussocks. Furthermore, at the intraspecific level, oothecae were placed shallower in bare ground or higher on tussocks with increasing local moisture and partially also with increasing temperature. In general, our results indicate that orthopterans optimise oviposition depth and even xeric orthopteran species prefer substrates with higher moisture to protect oothecae from desiccation.

Moisture is the most relevant factor determining successful egg development in insects^14^. Many species need to absorb water before embryogenesis begins, and eggs have to continuously resist desiccation until hatching occurs^21^. Almost all the investigated species laid at least some eggs into each moisture level (compartment), which suggests a relatively relaxed moisture preferences across orthopterans. Even in the most drought preferring species, *Chorthippus vagans* and *Stenobothrus crassipes*, females only slightly preferred the dry substrate over slightly wet or wet substrates, and some oothecae were laid in soaked soil. In general, wet soil was preferred the most and dry soil the least. Our findings provide support for the insurance hypothesis, which predicts a general tendency to oviposit into substrates with high moisture levels independently of the habitat preferred by adults^9,20,35^. In contrast, we did not find evidence supporting the environmental matching hypothesis, which predicts a correlation between species habitat preferences (xerothermophility) and oviposition preferences^7,18^. It seems that species do not detect moisture in an absolute manner, but only relatively in the context of occupied habitat. For example, the xerothermophilic species *Chorthippus mollis* strongly preferred soaked substrate in the experiment, despite almost never coming into contact with soaked soil in nature. *Omocestus viridulus*, which usually occurs in humid habitats, preferred tussocks within only slightly wet soil in our experiment. This can be an adaptation to spring flooding, which is common in its preferred habitats^21,36^.

The embryonic development rate is closely related to ambient temperature and the sum of degree days during a season can be a limiting factor for many temperate insects^32,37^. On the other hand, lethal temperature thresholds are usually very close to the thermal optima and a fine equilibrium between these two factors has to be reached^14,38^. Our data show that optima of all investigated species occurred between 29 and 34 °C, which fits well with previously published single-species data^21,25,31,39^. The relatively narrow range of preferred oviposition temperatures across species originating from variable habitats is quite surprising and seems to be quite conservative within the Gomphocerinae subfamily. In contrast to findings of Schnebel and Grossfield^13^, we observed no tendency of xerothermophilic species to prefer higher surface temperature for oviposition. However, Schnebel & Grossfield (1986) investigated fruit fly species along a latitudinal gradient ranging from arctic to tropic regions, not species from a single region. Again, a possible explanation of our findings can be based on individual life strategies of different species. For example, despite its high xerothermophility, *Chorthippus mollis* preferred the lowest oviposition temperature out of all investigated species. This species has a relatively long embryonic development and the choice of relatively cold and moist microhabitats within hot and dry steppes, where it lives, can protect developing immobile embryos from potentially damaging temperatures at the start of summer when other species have already hatched^32,36^. The fact that we measured surface temperature can potentially represent a methodological issue. In nature, as well as in our experiment, soil temperature decreases with distance from the surface. However, existing information on the oviposition site selection process in orthopterans indicates that ovipositing females perform a batch of shallow probes with their ovipositor, so surface conditions (including temperature) are probably crucial for their decision making^21^.

It has been hypothesized that xerothermophilic species should lay eggs deeper into the ground or lower within tussocks than oligothermic species to avoid damaging hot and dry conditions^7,21,40^. Our results partly supported this hypothesis, as tussocks-laid oothecae were placed closer to ground with increasing xerothermophility of species, and there was a similar, but non-significant tendency in bare ground-laid oothecae. In the case of phylogenetically-uncorrected models, significances were switched. This difference probably stems from accumulation of tussocks-laying species in the clade containing *Stenobothrus* and *Omocestus* genera (Fig. S4). In this case, results of phylogenetically-corrected models should be considered more relevant. Nevertheless, using more species, especially more hygro- and psychrophilic ones, in a future study will allow for a better evaluation of this pattern.

At the intraspecific level, we found a significant tendency to place oothecae deeper to the soil or closer to the ground within tussocks with decreasing moisture. A similar finding was previously reported for the grasshopper *Romalea microptera* in which females laid eggs shallower into the substrate when exposed to higher moisture levels^24^. Interestingly, the effect of temperature was surprisingly less pronounced and significant only for tussocks-laid oothecae. Phylogenetically-uncorrected models performed very similarly to corrected ones. This indicates that species traits and ecology are more important than evolutionary history of species in the case of intraspecific analyses.

We are aware of some potential methodological issues emerging from our experimental design, specifically those arising from possible interactions between animals. As described in methods, we isolated the cages as much as possible without affecting the environmental gradients within each cage. To reduce potential interactions between ovipositing females, we employed relatively large cages to avoid extremely high population densities, which were similar to those used in other laboratory studies^32,41,42^. Finally, although only one cage was used per species, each individual oothecae represents our data points and laboratory observations suggests that the choice of oviposition sites was not influenced by other individuals. Ideally, future studies should replicate each species in several cages if methodologically possible. In addition, oviposition preferences can vary between geographically distant populations within species and this phenomenon would also deserve a future attention.

Only a limited number of studies have investigated the effects of microclimate on oviposition behaviour compared to the wider range of published works that explore the effects of biotic factors, e.g., optimal food, parasitism, or competition avoidance^5,6^. Abiotic factors are directly modified by ongoing climate change and anthropogenic habitat alterations, which increases the relevance of such studies for nature conservation^16,43^. Moreover, eggs are an immobile life stage unable to escape from suboptimal conditions and frequently the most vulnerable to extreme temperatures^32,44^. This highlights the importance of studies investigating microhabitat oviposition preferences in insects. Especially valuable are studies employing a comparative framework, i.e., investigating a high number of species using a standardized experimental setting. Unfortunately, such studies are rare with the exception of a historical study of *Drosophila* flies^13^. However, our understanding of oviposition site selection cannot be complete without further studies investigating survival of eggs (oothecae) placed in various microhabitats and the related fitness consequences for emerging offspring. This represents a priority topic for future research focused on abiotic factors or interaction of abiotic and biotic factors affecting oviposition behaviour in insects.

## Materials and methods

### Specimen collection

Grasshoppers used for the laboratory experiment were collected between June 15th and July 15th, 2018, when most of Central European grasshopper species reach adulthood. All collecting sites were located within the Czech Republic. More specifically, xerothermophilic species were obtained from karst areas of Český kras and steppe habitats in České středohoří, hygro- and psychrophilic *Omocestus viridulus* were collected from Brdy Mountains, and the rest of the species were collected within the capital city of Prague. All sampled sites represented typical habitats for each species^36^. Grasshoppers were visually checked, and only freshly emerged adults (with a soft cuticle and deflated abdomen) or subadults were chosen for the laboratory experiment. This approach reduced a potential age effect on oviposition preferences^21^. Finally, a set of 14 grasshopper species with contrasting habitat requirements was included in the laboratory experiment (see Supplementary Table S2 for additional details on their biology and sampling sites).

### Experimental design

The laboratory experiment was performed in large cages (100 × 50 × 30 cm) made of 4 mm thick sheets of polycarbonate (bottom) and plastic mesh (walls). The bottom was divided lengthwise into four 100 × 12.5** cm** sized compartments, and each compartment was filled 5 cm thick substrate layer consisting of 1:1 peat and sand mixture. During the testing stage we repeatedly measured water content of substrate samples originating from various areas of large cages (measured gravimetrically). Our substrate ensured even distribution of moisture within each compartment as confirmed by a pilot experiment (testing stage). Based on various watering regimes tested during the testing phase we developed the appropriate final experimental setting. Compartments (moisture treatments) within a cage differed in their moisture levels as follows: (1) dry (approximately 5% moisture), (2) slightly wet (20%), (3) wet (35%) or (4) soaked (50%). The dry compartment did not get any extra water during the experiment. The slightly wet compartment was watered every other day with 200 ml, which resulted in transient wetness of the upper soil layer. The wet compartment was watered with 400 ml every other day to maintain permanent wetness across the whole soil profile. The soaked compartment received 600 ml every other day to reach fully saturated soil. A line of three 53 W halogen bulbs arranged 10 cm above the soil level was used to create a temperature gradient perpendicular to moisture gradient (Fig. 1). The function describing the relation of soil-surface temperature and the distance from the heat source was computed based on measurements performed across the cage length in 5 cm intervals, separately for each moisture compartment. Maximal surface temperature (42 °C) was reached directly under the heat source in the dry compartment. In the other compartments, maximal temperature gradually decreased with increasing moisture level (32 °C in the soaked compartment). Moreover, there was no other light source in the breeding room and cages were visually isolated from each other.

**Figure 5:**
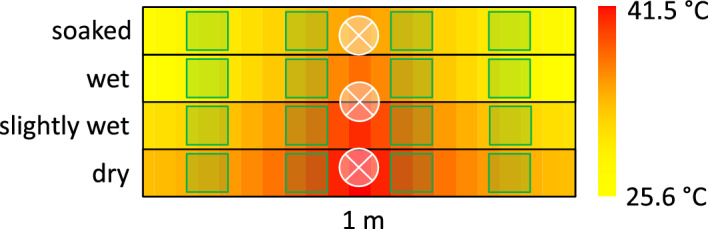
Scheme of our experimental cages (view from above). Background colours indicate local surface temperature, green squares represent grass tussocks, and crossed circles indicate positions of 53W halogen bulbs (placed 10 cm above the surface). Substrate humidity decreased from top to bottom. The bar on the right side shows the soil surface temperature recorded during the experiment.

Grasshopper species differ in their preferred oviposition substrate, some species preferring bare ground, others place eggs (oothecae) into grass roots or grass tussocks, and some species use both strategies^21^. Therefore, four grass tussocks (ca. 10 × 10 cm) were planted in each compartment on both sides of the heat source at a distance of 12 and 35 cm (see the cage setup in Fig. 5).

Twenty females and 15 males per species were released in each cage and each species had its own cage (i.e., 14 cages were used in total). Similar population densities were used in various laboratory studies on Central European gomphocerinae grasshoppers^32,41,42^. However, natural population densities are probably lower. Cages were maintained until the death of the last female, typically for two or three months. Grasshoppers were provided with food in the form of a mixture of grasses placed in water-filled plastic tubes. Several tubes were dispersed across each cage and grasses were changed every three days or when depleted. Cages were kept at a L16 : D8 photoperiod and ambient temperature fluctuating between 26 ± 2 °C during the light period and 17 ± 2 °C during the dark period. Ambient humidity ranged 40–60%. All investigated species are strictly day-active^21^ and it is expected that oviposition took place solely during the daytime.

After the death of the last female, each cage was carefully inspected for oothecae. For each ootheca, the substrate (bare ground vs. tussock), the compartment moisture level (dry, slightly wet, wet, soaked), the vertical position relative to the soil surface (the depth in the soil, or the height above the ground in the case of an oviposition on grasses), and the distance from the heat source (to calculate temperature) were recorded. The vertical position of ootheca was measured as the position of its centre with a precision of ± 0.5 cm.

To minimize possible effects of neighbouring cages on experimental animals we tried to isolate the cages from each other as much as possible (at least one meter apart) and keep the same gradients within each cage. We also eliminated all outside sources of light. To reduce potential interactions between ovipositing females, we used relatively large cages (100 × 50 × 30 cm), reducing population densities. In addition, ovipositing females were observed ca. 10 h during the initial stage of the experiment and for several hours during following weeks and no interactions between ovipositing females were detected. Furthermore, we did not recorded any strange pattern in spatial distribution of oothecae (e.g., small-scale nesting or even distribution across the cage), indicating only negligible interactions between ovipositing females.

### Data analysis

#### Phylogenetic data

To address phylogenetic relationships between investigated species, we constructed a phylogenetic tree following methods and data of the most recently published phylogeny^45^ and added an additional COI sequence of *Stenobothrus crassipes*, which was obtained from the BOLD database (^46^; BOLD: AAD9833). The final phylogenetic tree is included in supplementary materials (Fig. S4). Four genes were used: two mitochondrial—Cytochrome C oxidase subunit I (COI, 575 bp) and Cytochrome B (cytB, 579 bp) and two nuclear—Internal transcribed spacers 1 and 2 (ITS1, 395 bp and ITS2, 293 bp). However, note that for particular species not all the above mentioned gene sequences are available (for details on other species than *Stenobothrus crassipes* see the original study^45^). Acquired sequences were aligned and trimmed in Aliview 1.28 software^47^. The Bayesian inference method (MCMC) was used to create phylogenetic trees and we performed a BEAST analysis (BEAST 2.7.4^48^). Evolutionary models were fitted using BEAUti 2.7.4 software^48^. A fixed clock model was used to estimate divergence time. Analysis was set to 20 million generations run and sampling step 2000 generations. Finally, the TreeAnnotator 2.7.4 software^48^ (with 10% burn-in) was used to compute consensus tree and estimate the posterior probabilities (PP).

#### Moisture and temperature indices

To describe species oviposition preferences, we assessed the most preferred level (mode) and mean of moisture (dry, slightly wet, wet or soaked), the mean laying depth (vertical position according to the soil surface), and the mean preferred soil surface temperature. To test the existence of specific preferences for moisture, chi-squared tests were used for each species separately. Our null hypothesis assumed the random distribution among moisture compartments, i.e., 25% probability for each compartment. Significant differences between species oviposition temperature and vertical position were tested with a One-way ANOVA. Only species producing more than 10 oothecae per substrate type were included in our analyses.

To investigate the relationship between the index of xerothermophility (developed in our previous study; Table 1; for details see^30^) and species-specific microclimatic preferences, several phylogenetic linear mixed-effect models were fitted. Separate models were used for investigated response variables (moisture, temperature, vertical position of oothecae) and substrate type (bare ground, tussocks), i.e., in total six models were fitted. Index of xerothermophility was used as fixed effect and species identity as random effect in all models. To account for phylogenetic relations, we used “Almer” extension for “lme4” package^49^ and the newly constructed phylogenetic tree (see above). For analyses of the vertical position of oothecae, the contribution of each species had to be weighted by the ratio of bare ground-laid or tussock-laid oothecae as records of rare oothecae in non-preferred substrate type could represent problematic cases. To investigate the importance of phylogenetic corrections, we also fitted phylogenetically-uncorrected version of all models (GLMMs, using “lmer” function).

Finally, we investigated the effects of microclimatic conditions on the intraspecific vertical position of oothecae using phylogenetic linear mixed-effect models in which the vertical position of ootheca was the response variable. Moisture, temperature, and their interaction were used as fixed effects. Species identity was included as a random effect. Two separate models were fitted for bare ground-laid and tussock-laid oothecae. We also fitted phylogenetically-uncorrected versions of models for comparison. All analyses were performed in R 3.5.2^50^.

### Supplementary Information

Supplementary Information 1.

Supplementary Information 2.

Supplementary Table S1.

## Data Availability

All data generated or analysed during this study are included in its supplementary information files.
