# Supplementary material for: Conserved temperature requirements but contrasting responses to humidity across oviposition preferences in temperate grasshoppers
Source: Sci Rep. 2023 Nov 30;13:21131. doi: 10.1038/s41598-023-47789-z (PMC10689742; doi:10.1038/s41598-023-47789-z)
Supplement: Supplementary file 2 — Supplementary Information 2. [file 41598_2023_47789_MOESM2_ESM.pdf]

Oviposition preferences in temperate grasshoppers: Conserved temperature requirements but contrasting responses to humidity across species

Tomáš DVOŘÁK [1, 2], Michal KNAPP [2]

[1] *Department of Zoology, Faculty of Science, Charles University, Viničná 7, Prague 2, 128 44, Czech Republic*

[2]*Department of Ecology, Faculty of Environmental Sciences, Czech University of Life Sciences Prague, Kamýcká 129, Prague – Suchdol, 165 00, Czech Republic*

Supplementary Materials

Figure S1: Species specific vertical position of oothecae. Boxes show second and third quartile limits, solid lines represent first and fourth quartile limits and dots represent outliners. Black lines inside a boxes represent median values. Zero indicates the substrate surface, negative values represent buried oothecae, or oothecae placed into roots and positive values represent oothecae placed on blades of grasses.

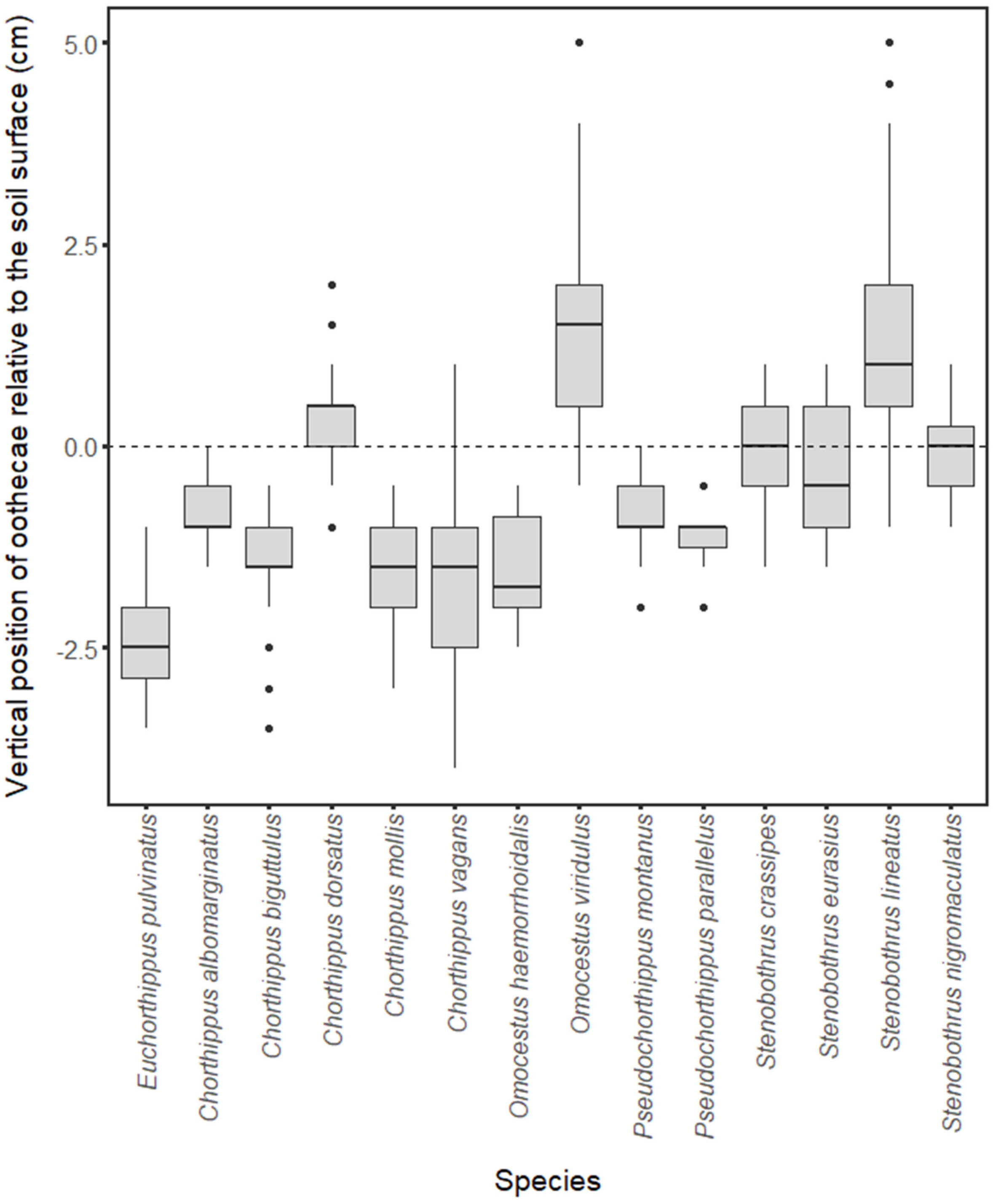

Figure S2: Effects of moisture on species specific vertical position of bare ground-laid oothecae. Full line represents the overall fit (across all species) based on the phylogenetically corrected mixed-effect model. In general, increasing substrate moistures resulted in significantly shallower placement of oothecae ($p < 0.001$).

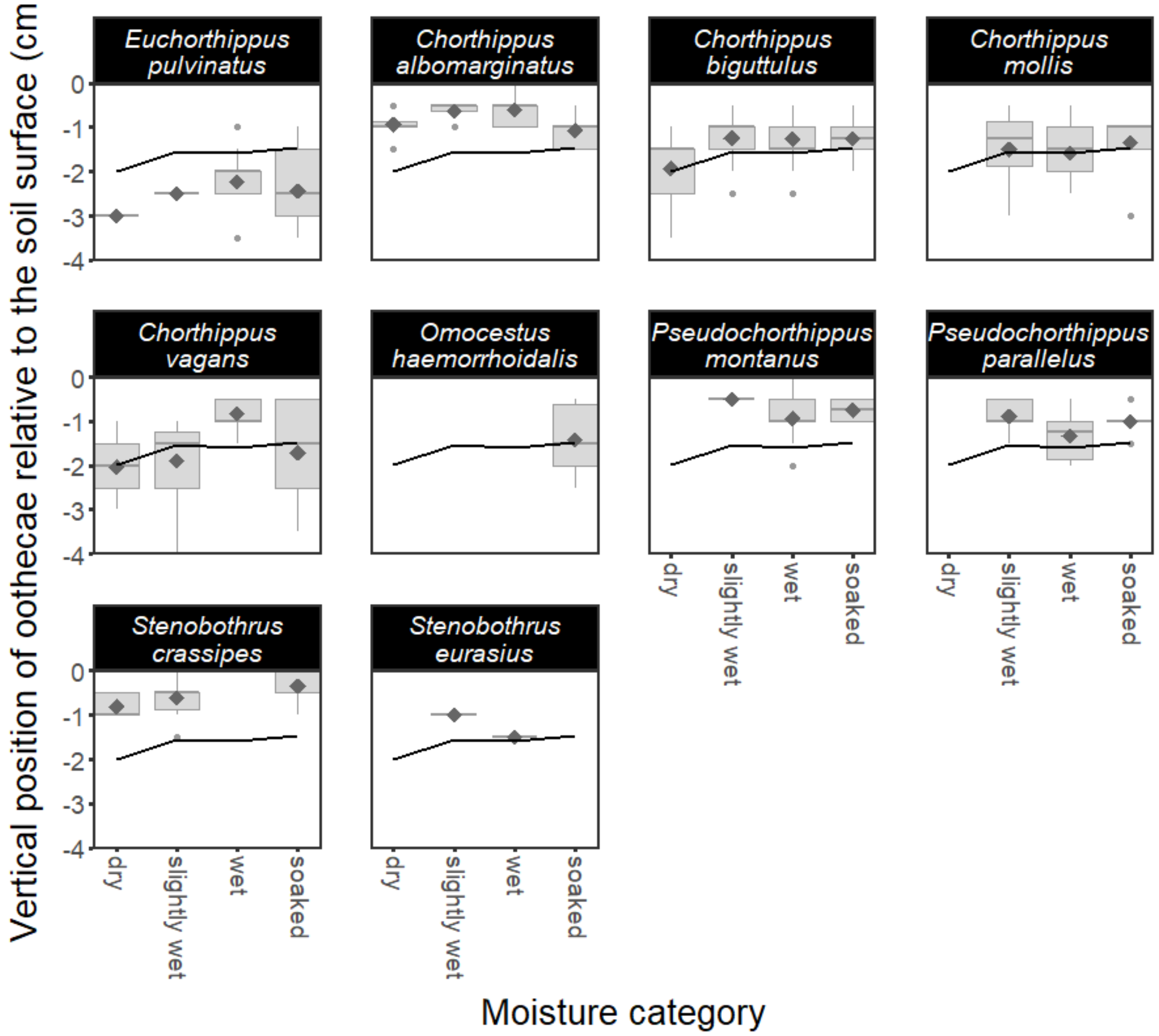

Figure S3: Effects of moisture on species specific vertical position of tussock-laid oothecae. Full line represents the overall fit (across species) based on the phylogenetically corrected mixed-effect model. In general, vertical position of oothecae significantly increased with substrate moisture ($p < 0.001$).

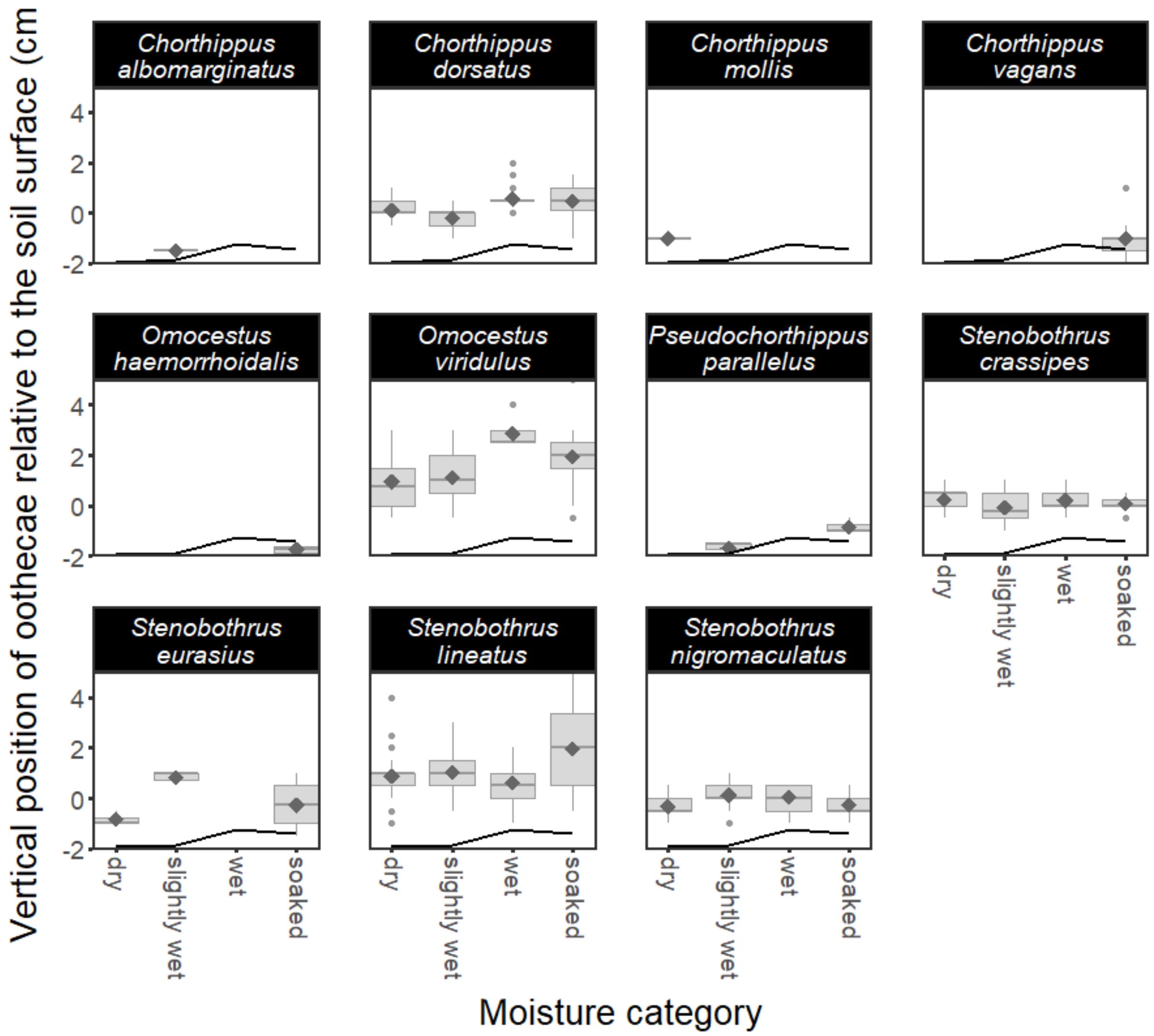

Figure S4: Phylogenetic tree of species investigated in the present study including *Locusta migratoria* as the outgroup. The scale bar below the phylogenetic tree shows branches age in million years. Note the position of *Stenobothrus crassipes*, which has not been yet included in any previous published phylogeny. Evolution of oviposition behaviour (proportion of tussocks-laid oothecae) is visualised using colours (see the figure legend for details). Note that the ground-laying strategy represents the ancestral state.

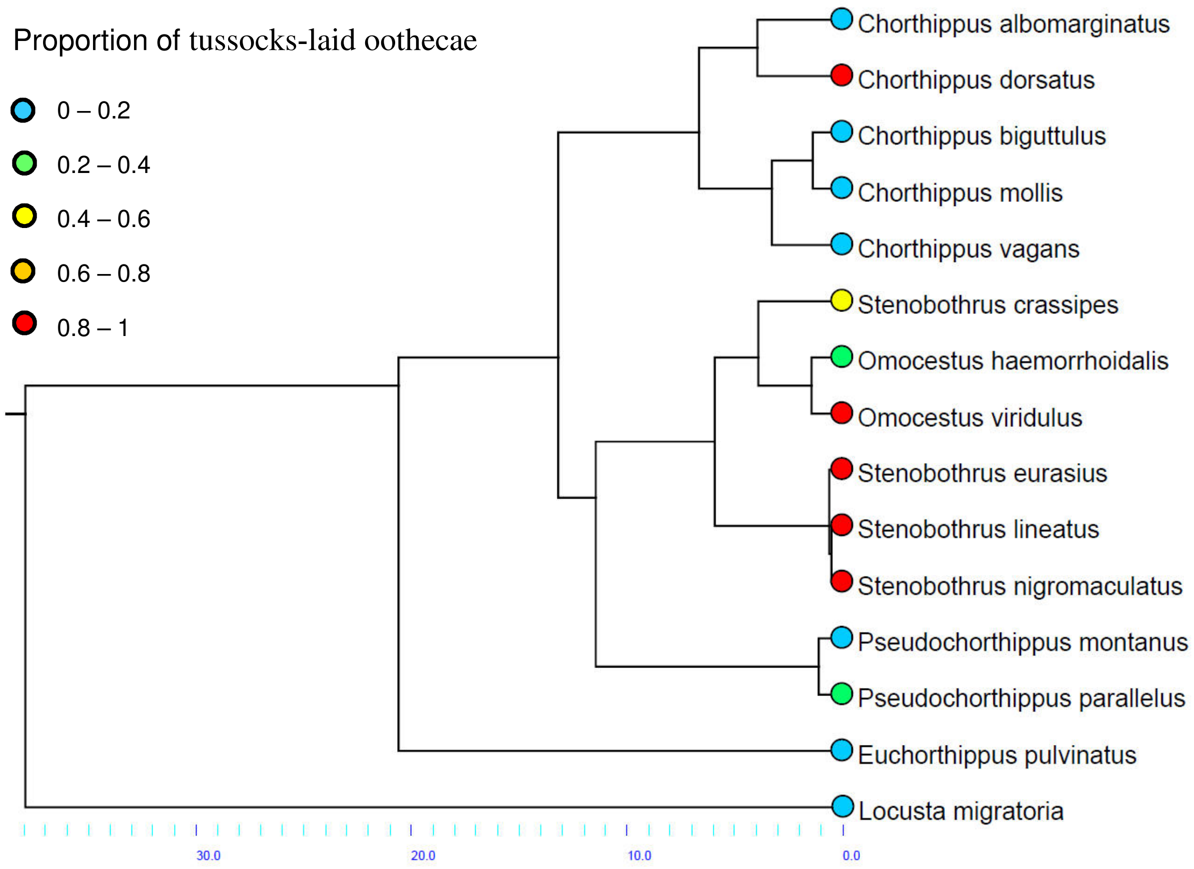

Table S2: Detailed description of species habitat, distribution in Central Europe and oviposition substrate obtained from literature. Xerothermophility index and photo is also included.

Species	Photo	Xerothermophility index[1]	Habitat description	Collection site	Oviposition substrate [2,3]	Distribution in Central Europe
Euchorthippus pulvinatus	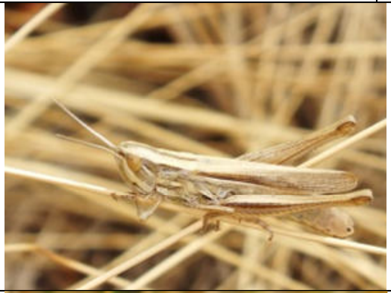	0.896	Steppe with relatively high but sparse vegetation	*Stipa* spp. dominated steppes in Raná hill in České středohoří, Czech Republic	Soil	Rare, Czech Republic is the northwestern limit of its range
Chorthippus albomarginatus	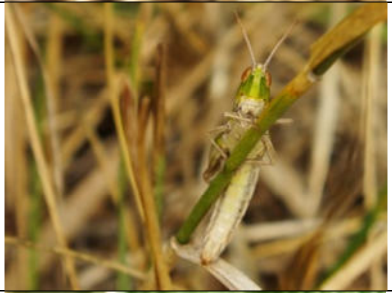	0.336	Moderately wet to wet meadows and pastures, prefers lower vegetation	meadows near Šeberov, Prague, Czech Republic	Base of grass tuft	Abundant from lowlands to lower mountains
Chorthippus biguttulus	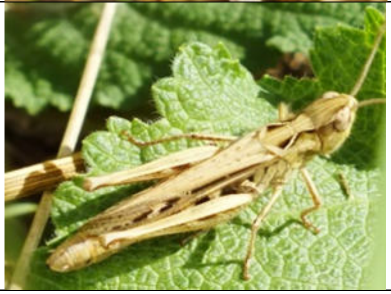	0.518	Moderately wet to dry meadows, pastures, steppes; avoids too high and dense vegetation	meadows near Šeberov, Prague, Czech Republic	Soil	Very abundant from lowlands to lower mountains

Chorthippus dorsatus	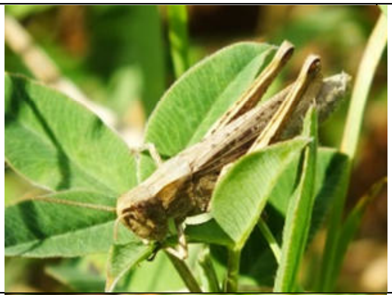	0.375	From slightly dry to wet meadows, tolerates ruderalised high vegetation	meadows near Šeberov, Prague, Czech Republic	Base of grass tuft and into roots	Abundant from lowlands to lower mountains
Chorthippus mollis	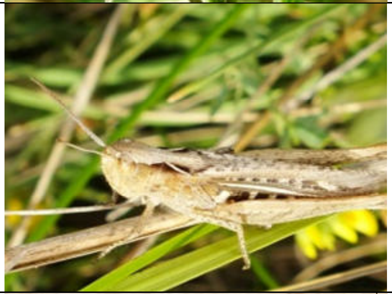	0.900	Steppes, rocky steppes, dry meadows and pastures with shorter and sparse vegetation	steppes and dry pastures near Radotín, Prague, Czech republic	Soil	Quite rare, can be locally abundant in lowlands
Chorthippus vagans	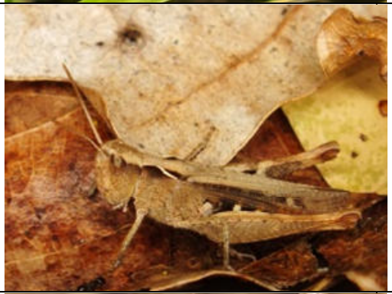	0.960	Dry forest clearings and edges, forest steppe, rocky steppe, prefers minimal vegetation cover	forest clearings in Kunratice, Prague, Czech Republic	Soil	Quite rare, occurs from lowlands to hills
Omocestus haemorrhoidalis	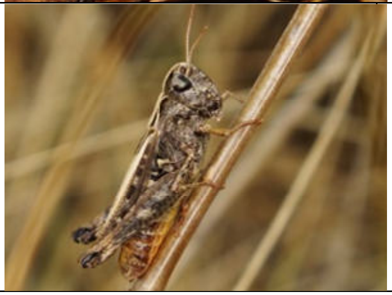	0.799	Dry meadows and pastures, heaths, sandy areas; occupies moderately high and not too dense vegetation	Heaths in Troja, Prague, Czech Republic	Soil	Locally abundant, from lowlands to lower mountains

Omocestus viridulus	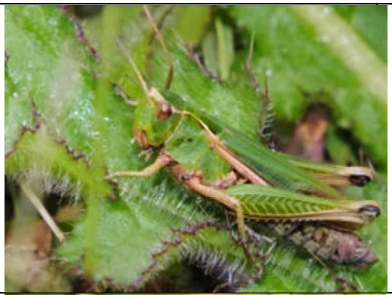	0.109	From moderately wet to wet meadows, alpine grasslands, peat bogs; prefers higher altitudes	Wet meadows near Voznice, Brdy mountains, Czech Republic	Base of grass tuft	Locally abundant, mostly in mountains
Pseudochorthippus montanus	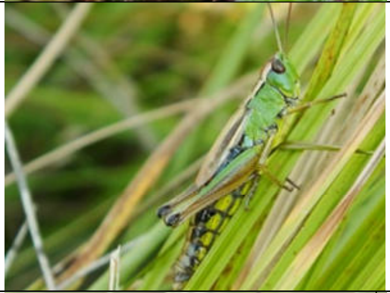	0.021	Wet meadows, sedges	wet meadows near Háje, Prague, Czech Republic	Soil	Locally abundant from lowlands to lower mountains
Pseudochorthippus parallelus	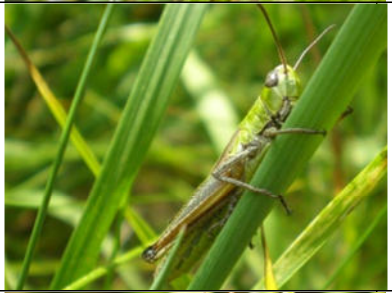	0.291	Broad ecological niche, from dry to wet grasslands, only avoid extremely sparse and extremely dry vegetation	meadows near Šeberov, Prague, Czech Republic	Soil	Very abundant from lowlands to mountains
Stenobothrus crassipes	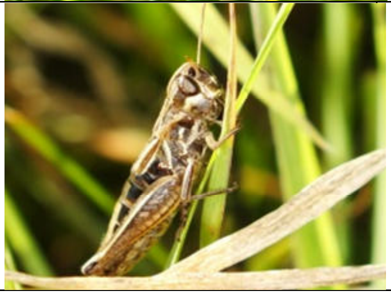	0.780	Moderately dry steppes and meadows without too high and dense vegetation	steppes and dry pastures near Radotín, Prague, Czech Republic	Base of grass tuft	Rare, more abundant only in lowlands

Stenobothrus eurasius	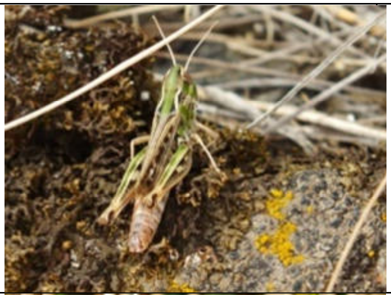	1	Extremely dry and exposed rocky steppes with very sparse vegetation cover	Rocky steppes in Oblík hill in České středohoří, Czech Republic	-	Very rare, Czech Republic is the northwestern limit of its range
Stenobothrus lineatus	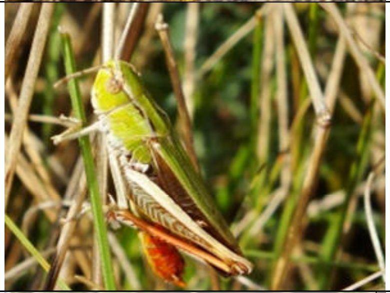	0.777	Dry meadows and meadow steppes with not too high vegetation	steppes and dry pastures near Radotín, Prague, Czech republic	Base of grass turf, into roots or soil	Locally abundant from lowlands to lower mountains
Stenobothrus nigromaculatus	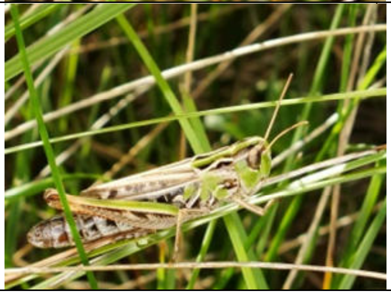	0.952	Diverse steppes, needs sparse and short vegetation cover	steppes and dry pastures near Radotín, Prague, Czech Republic	Base of grass turf, into roots or soil	Rare, more abundant only in lowlands

1. Dvořák, T., Hadrava, J. & Knapp, M. *Biological Conservation* **265**, 109406 (2022).
2. Ingrisch, S. & Köhler, G. *Die Heuschrecken mitteleuropas*. (Die Neue Brehm-Bücherei, 1998).
3. Kočárek, P., Holuša, J., Vlk, R. & Marhoul, P. *Rovnokřídlí České republiky*. (Academia, 2015).
